# In Vitro Probiotic and Antioxidant Potential of *Lactococcus lactis* subsp. *cremoris* LL95 and Its Effect in Mice Behaviour

**DOI:** 10.3390/nu11040901

**Published:** 2019-04-22

**Authors:** Juliana B. Ramalho, Melina B. Soares, Cristiano C. Spiazzi, Diogo F. Bicca, Vanessa M. Soares, Juliano G. Pereira, Wladimir P. da Silva, Carla P. Sehn, Francielli W. S. Cibin

**Affiliations:** 1 Laboratório de Biotecnologia da Reprodução (Biotech), Universidade Federal do Pampa (UNIPAMPA), Campus Uruguaiana, Uruguaiana 97501-970, RS, Brazil; julianabramalho@gmail.com (J.B.R.); melina_bucco@hotmail.com (M.B.S.); cris.spiazzi@gmail.com (C.C.S.); diogofb5@hotmail.com (D.F.B.); 2 Programa de Pós-Graduação em Ciência Animal, PNPD/CAPES, Universidade Federal do Pampa (UNIPAMPA), Campus Uruguaiana, Uruguaiana 97501-970, RS, Brazil; vanessamsoares@gmail.com; 3 Departamento de Higiene Veterinária e Saúde Pública, Faculdade de Medicina Veterinária e Zootecnia. Universidade Estadual Paulista (UNESP), Campus de Botucatu, Botucatu 18618-681, São Paulo, Brazil; juliano.pereira@unesp.br; 4 Departamento de Ciência e Tecnologia Agroindustrial (DCTA), Universidade Federal de Pelotas (UFPel), Pelotas 96010-900, RS, Brazil; silvawp@ufpel.edu.br; 5 Laboratório de Avaliações Farmacológicas e Toxicológicas Aplicadas às Moléculas Bioativas (LaftamBio), Universidade Federal do Pampa (UNIPAMPA), Campus Itaqui, Itaqui 97650-000, RS, Brazil; carla.pohlsehn@gmail.com

**Keywords:** *Lactococcus lactis* subsp. *cremoris*, probiotic, antioxidant activity, depression, anxiety

## Abstract

The composition of intestinal microbiota is widely believed to not only affect gut health but also influence behaviour. This study aimed to evaluate the probiotic characteristics, antioxidant activity, and antidepressant- and anxiolytic-like activities of *Lactococcus lactis* subsp. *cremoris* LL95. This strain showed probiotic properties such as resistance in a simulated gastric tract model and survival at different concentrations of NaCl and bile salts. Moreover, antioxidant activity of LL95 was demonstrated through DPPH radical scavenging activity, scavenging of ABTS^•+^ radical and ferric ion reducing antioxidant power (FRAP) assays. Female C57BL/6 mice received LL95 orally at a dose of 10^9^ UFC/day for 28 days. LL95 improved depressive- and anxiety-like behaviour, demonstrated by decreased immobility time in the tail suspension test and forced swim test and increased per cent of time spent in the open arms on the elevated plus maze. These findings indicate the potential antioxidant activity of LL95 and its role in behaviour, suggesting that probiotic may have therapeutic applications.

## 1. Introduction

The microbiota that inhabits the human gut is part of an extremely complex ecological system. These microorganisms interact not only with each other, but also with their host in a symbiotic relationship [1]. The imbalance in the composition of gut microbiota, a condition known as dysbiosis, has negative health consequences. Several diseases have been linked to dysbiosis, including atopic dermatitis, inflammatory bowel disease, diabetes, obesity, cancer and, recently, neuropathology [2,3].

Due to the importance of gut microbiota in health maintenance and disease prevention, there is a growing interest in strategies capable of their modulation, for example through the ingestion of probiotics [4]. Probiotics are live microorganisms that, when administered in adequate amounts, confer health benefit to the host [5]. Lactic acid bacteria (LAB) and bifidobacteria are the most common types of microorganisms used as probiotics, although other bacteria and certain yeasts are also used [6]. The most important strain properties to even be considered for probiotic use include resistance to gastric acidity, bile acid resistance, antimicrobial activity against potentially pathogenic bacteria or fungi and ability to reduce pathogen adhesion to surfaces [7]. In vitro studies provide the first step in evaluating probiotics and these tests ensure that the microorganism is able to withstand the conditions of gastrointestinal tract and exert their functional properties [8]. 

There is a growing body of evidence demonstrating an interaction between the intestinal microbiota, the gut and the central nervous system (CNS), known as the brain–gut–microbiota axis [9]. This link between the gut microbiota and CNS suggests that their modification may have applications in the treatment of neuropsychiatric disorders [10,11,12]. Several preclinical studies have evaluated whether manipulation of the gut microbiota through the ingestion of probiotics can affect behaviour in animals. They have shown that probiotics may be used to modulate behaviours related to psychiatric disorders, including depressive-like behaviour [10,13,14,15,16,17,18]. However, the mechanisms underlying the ability of certain bacteria to modulate depressive-like behaviour remain to be elucidated. 

Depression is a heterogeneous disorder with a highly variable course, and, according to the World Health Organization (WHO), it must become the biggest disabling disorder in the next 20 years [19]. Furthermore, the comorbidity of depressive disorders and anxiety is common and individuals affected by both disorders concurrently have shown reduced quality of life, and poorer treatment outcomes compared with individuals with only one disorder [20]. Currently, most pharmacological treatments for depression focus on neurotransmitter deficiency in monoaminergic synapses. However, such drugs have delayed action and may exhibit adverse side effects such as headaches, nausea, agitation, or sedation [21]. Therefore, the search for new therapeutic agents has been increasingly stimulated.

*Lactococcus* is a genus of Gram-positive LAB and the species *Lactococcus lactis* is commonly used as starter culture in the manufacture of fermented dairy products such as cheese and yogurt [22]. Certain strains of *Lactococcus lactis* subsp. *cremoris* have shown beneficial effects in animal models. Its protective action has already been demonstrated against changes induced by carbon tetrachloride, against the influenza virus and in sodium dextran sulphate-induced colitis [23,24,25,26]. It is important to note that the biological effects of probiotics are strain-specific and that the success of one strain cannot be extrapolated to another strain [27].

Therefore, the objective of this study was to evaluate the probiotic characteristics and the antioxidant activity in vitro of *L. lactis* subsp. *cremoris* LL95, a LAB isolated from cheese. In addition, the safety and the possible antidepressant- and anxiolytic-like activity of this strain in mice were investigated. 

## 2. Materials and Methods

### 2.1. Drugs

Ascorbic acid, 2,4,6-Tris(2-pyridyl)-s-triazine (TPTZ) and 2′,7′-dichlorodihydrofluorescein diacetate (DCFH-DA) were purchased from Sigma-Aldrich (St. Louis, MO, USA). All other chemicals were of analytical grade and obtained from standard commercial suppliers.

### 2.2. Bacterial Strain and Growth Conditions

*Lactococcus lactis* subsp. *cremoris* LL95 was obtained from the culture collection of the Food Microbiology Laboratory—Department of Agroindustrial Science and Technology of the Federal University of Pelotas (UFPel), Brazil. This strain was isolated from sliced mozzarella cheese sold in markets and minimarkets in Pelotas, Rio Grande do Sul, Brazil.

Sourced from frozen stocks (−80 °C), bacteria were reactivated in De Man, Rogosa and Sharpe broth (MRS broth) (Merck^®^, Darmstadt, Germany), plated in MRS agar, cultured anaerobically at 37 °C for 24 h, then inoculated in fresh MRS broth and grown at 37 °C under aerobic conditions for 24 h in 250 mL Erlenmeyer flasks containing 50 mL of MRS medium. 

### 2.3. In Vitro Evaluation of Probiotic Characteristics

#### 2.3.1. Survival in Simulated Gastrointestinal Tract Conditions

The analysis of the survival of bacteria in simulated gastrointestinal tract conditions was performed as reported by Huang and Adams [28]. Briefly, the culture was carried out in 5 mL of MRS broth and incubated at 37 °C for 24 h, followed by centrifugation at 6800× *g* for 10 min at 4 °C. The obtained pellet was washed twice with phosphate buffer saline (PBS), then resuspended in 0.5% saline. A 200 μL aliquot of the cell suspension was mixed in 300 μL of saline and 1 mL of gastric juice, with subsequent incubation at 37 °C. The simulated gastric juice consisted of 3 mg/mL pepsin (Sigma-Aldrich^®^, St. Louis, MO, USA) pH 2.5. The viable cell counts were performed initially and later at 15, 30, 60, 120, 180 and 240 min to evaluate tolerance to simulated gastric juice in plates containing MRS agar (Merck^®^) and incubated at 37 °C after 72 h. The effect of the presence of food on the survival during simulated transit in gastric juice at pH 2.5 was evaluated under the same conditions except that the saline was substituted with 10% (*w*/*v*) skimmed milk.

#### 2.3.2. Evaluation of The Resistance to Different Concentrations of NaCl, pH and Bile Salts

The ability of bacteria to multiply at different concentrations of NaCl, pH and bile salts was analysed, as reported by Drosinos et al. [29], with some modifications. The isolate was cultured in MRS broth (Merck^®^, Darmstadt, Germany) and subsequent passage (1% v/v) of the culture for the different conditions. To test bacterial growth in the presence of salt, pH and bile salts, the MRS broth was modified with 4.5% and 6% of NaCl (Synth^®^, Diadema, Brazil), pH 2.0 (HCl) and the addition of bovine bile at 0.3% and 1% (v/v), respectively. The isolate was incubated at 37 °C for 24 h. After incubation, the cultures were transferred to agar MRS and incubated at 37 °C. The presence of growth in the medium after 24 h of incubation was considered evidence of the ability of bacteria to support the different conditions.

#### 2.3.3. Autoaggregation and Coaggregation Abilities

The autoaggregation and coaggregation abilities of the bacteria were evaluated according to the methodology described by Collado, Meriluoto and Salminen [30]. Cell suspension was obtained from the culture of the isolate in MRS broth and the indicator microorganism in Brain Heart Infusion (BHI) (Himedia^®^, Mumbai, India) at 37 °C for 24 h. The culture was centrifuged at 6800× *g* for 10 min at 4 °C and the pellet was washed twice with PBS. Then, the cells were resuspended in 0.5% saline and absorbance at 600 nm was adjusted to 0.25 ± 0.02 for the tests.

Autoaggregation was determined by reading the absorbance (600 nm) of the cell suspensions, termed as the total bacterial suspension (time zero), and after 2, 20, and 24 h of incubation at 20 °C and 37 °C, termed as the upper suspension. The autoaggregation percentage was expressed as: autoaggregation (%) = (1 − A upper suspension/A total bacterial suspension) × 100.

To determine the coaggregation with *Listeria monocytogenes* ATCC 7644, cell suspensions were prepared as described above, incubated at 20 °C and at 37 °C alone (controls), with equal proportions of *L. lactis* LL95 and the indicator microorganism (1:1). Absorbance readings (600 nm) were taken after 0, 2, 20, and 24 h of incubation. The results were expressed in percentages as follows: coaggregation (%) = [(At0) − (Atx)/(At0)] × 100, where At0 represents the absorbance of the bacterial suspensions at initial time (zero) and Atx represents the absorbance of bacterial suspensions at the different times tested.

#### 2.3.4. Antimicrobial Activity

The antimicrobial activity of *L. lactis* LL95 was tested by the spot-on-the-lawn technique, as described by Biscola et al. [31], with minor modifications, against *Escherichia coli* O157:H7 ATCC 43895, *Klebsiella pneumoniae* CCBH6603, *Listeria monocytogenes* ATCC 7644 and *Staphylococcus aureus* ATCC 25923. Briefly, aliquots of 2 μL from 24 h at 37 °C culture of *L. lactis* LL95 were spotted onto MRS agar after 24 h of incubation under anaerobic conditions at 37 °C. Then, the plates were covered with a semisolid BHI (Himedia^®^, Mumbai, India) agar containing 10^5^ CFU/mL of the indicator microorganism and incubated for 24 h at 37 °C. The plates were then analysed for the presence or absence of inhibition halos (transparency zones around the isolate). The presence of this indicated antimicrobial activity and the diameters were measured using a calliper (Digimess^®^, São Paulo, Brazil). 

#### 2.3.5. Antibiotic Susceptibility Tests

To evaluate phenotypic antibiotic susceptibility, the agar disc diffusion method was applied using MRS agar. The test was performed as described by the European Food Safety Authority [32]. Twelve antibiotics were tested: amikacin (30 μg), ampicillin (10 μg), chloramphenicol (30 μg), erythromycin (15 μg), gentamicin (10 μg), penicillin G (10 μg), trimethoprim-sulphamethoxazole (25 μg), tetracycline (30 μg), vancomycin (30 μg), clindamycin (2 μg), ciprofloxacin (5 μg) and sulphonamide (300 μg). The plates were incubated for 24 h at 37 °C. The diameters of the zones of inhibition were measured using a Digimess^®^ calliper and the results were expressed according to Liasi et al. [33] as follows: resistant (≤ 15 mm), intermediate (16–20 mm) or sensitive (≥ 21 mm). 

### 2.4. Determination of In Vitro Antioxidant activities of *L. lactis* subsp. *cremoris* LL 95

#### 2.4.1. Preparation of Intact, Heat-Killed, and Lyophilised Cells

*Lactococcus lactis* LL95 was incubated in MRS broth overnight at 37 °C and intact cells were harvested by centrifugation (10000× *g*, 10 min) at 4 °C. The resulting pellets were washed twice with sterile PBS and resuspended in sterile distilled water at a final concentration 10^9^ CFU in 25, 100 or 1000 µL, according to the test to be performed. Heat-killed cells were obtained by placing the samples in a water bath at 95 °C for 30 min [34]. To obtain lyophilised cells, the samples were dehydrated by freeze-drying (Liotop, Model-L101, São Carlos, Brazil) for 24 h. 

#### 2.4.2. DPPH Radical Scavenging Activity Assay

The DPPH radical scavenging assay was performed according to Xing et al. [35]. Briefly, 1 mL of LL95 was mixed with 1 mL of DPPH solution (0.2 mM). The mixture was incubated in the dark at room temperature for 30 min. Distilled water was used for the control sample. The scavenged DPPH was then monitored by determining the absorbance at 517 nm. The radical scavenging activity was quantified using the following formula: DPPH radical scavenging activity (U/mL) = ABS_C_ − ABS_S_/S × 100, where ABS_C_ and ABS_S_ are the absorbance of the control and the test samples at 517 nm, respectively, and S is the volume (mL) of the sample. The results are expressed as DPPH radical scavenging activity (U/mL).

#### 2.4.3. Scavenging of ABTS^•+^ Radical

The ABTS^•+^ radical scavenging assay was performed according to Han et al. [36]. Briefly, the ABTS^•+^ was diluted in a 20 mM sodium acetate buffer (pH 4.5). Posteriorly, 100 µL of LL95 samples were mixed with 3 mL of ABTS^•+^ solution and incubated in the dark at room temperature for 6 min. The absorbance of mixture was measured at 734 nm, calculated as follows: ABTS^•+^ scavenging rate (%) = (Ac − As)/Ac × 100, where A_S_ is the absorbance of the *L. lactis* LL95 sample and A_C_ is the absorbance of control (ABTS^•+^ solution without LL95 sample). The results are expressed as ABTS^•+^ scavenging rate (%).

#### 2.4.4. Ferric Ion Reducing Antioxidant Power (FRAP)

The reducing power of LL95 was tested by the FRAP assay according to Han et al. [36] with minor modifications. Briefly, 750 µL of FRAP solution was heated to 37 °C in a water bath. Then, 25 µL of sample and 75 µL of ultrapure water were added. The mixture was placed in the dark for 5 min and the absorbance was measured at 593 nm. A standard curve was prepared with 0.1, 0.2, 0.4, 0.6, 0.8 and 1.0 mM FeSO_4_.7H_2_O. The FRAP values of samples were calculated according to the standard curve. The results were expressed as the equivalent amount of FeSO_4_ (mM).

### 2.5. In Vivo Experiments

The first step of knowledge on probiotic strains is conducted through in vitro assays. However, health benefits of probiotics can only be demonstrated by in vivo studies. Animal models can be used to determine the safety of probiotic microorganisms, characterise a possible mechanism of action, or convey other knowledge of probiotic strains. In this way, the in vivo safety and possible antidepressant- and anxiolytic-like effects of L. lactis subsp. cremoris LL95 in mice were evaluated.

#### 2.5.1. Animals

The in vivo experiments were conducted using female C57BL/6 mice (20–22 g, 90 days old). Animals were maintained in an appropriate cabinet with forced air ventilation, under a 12-h light/dark cycle, at a controlled room temperature of 22 ± 2 °C, with food and water provided ad libitum. This study was approved by the Ethics Commission for Animal Use of the Federal University of Pampa (CEUA protocol #028/2017) and the procedures used were conducted according to the guidelines of the Committee on the Care and Use of Experimental Animal Resources. This study was developed in female mice given the susceptibility of women to the development of mood disorders [37]. 

#### 2.5.2. Experimental Procedure

**Figure 1:**
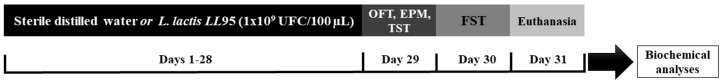
Experimental procedure. Abbreviations: OFT, Open-Field Test; EPM, Elevated Plus Maze; TST, Tail Suspension Test; FST, Forced Swim Test.

Ninety-day-old female mice received gavage with 100 μL of sterile distilled water (control group, *n* = 6) or a daily dose of 1 × 10^9^ CFU (suspended in 100 μL sterile distilled water, once a day) freeze-dried *L. lactis* LL95 (LL95 group, *n* = 6). This procedure was carried out daily for a period of 28 continuous days [10]. At the end of the treatment, the animals underwent a series of behavioural testing, including open-field, elevated plus maze, tail suspension, and forced swim tests (Figure 1).

#### 2.5.3. Antidepressant- and Anxiolytic-Like Evaluation

The behavioural tests were performed from the least stressful to the most stressful, to avoid stress interference in the results. The tests were recorded using a video camera supported by a tripod and the images were analysed using ANY-maze Video Tracking System version 6.06 (Wood Dale, USA).

##### Open-Field Test (OFT)

On Day 29, the mice were introduced to an open-field apparatus (50 cm × 50 cm) made of plywood with the floor divided into sixteen squares [38]. Each animal was placed individually at the centre of the apparatus and their behaviour was monitored for 5 min. This test was carried out to assess the possible effect of the treatments on locomotor activity and to measure anxiety-like behaviour. 

##### Elevated Plus Maze (EPM)

The EPM apparatus consisted of a plus-shaped made of plywood with two opposing open arms (30 cm × 8 cm) and two opposing closed arms (30 cm × 8 cm × 15 cm) connected by a central platform (8 cm × 8 cm) and elevated 120 cm above the floor. Each mouse was placed in the central platform, facing an open arm. The total arm entries and the amount of time spent by animals in the open and closed arms of the maze was measured during a 5-min period. The number of total entries, per cent of open entries (open entries/total entries × 100) and per cent of time in the open arms (time in open arms/total time of the test × 100) were determined. A reduction of the percentage of time spent and the number of entries into the open arms was considered as an anxiety-like index, independent of locomotor activity [39].

##### Tail Suspension Test (TST)

The TST was carried out according to Steru et al. [40]. In this test, mice were suspended 50 cm above the floor by adhesive tape placed approximately 1 cm from the tip of their tail. During a 6-min test period, the immobility time was scored for the last 5 min following a 1-min habituation period. The mice were considered immobile only when they hung passively and completely motionless. 

##### Forced Swim Test (FST)

The FST was performed according to Porsolt et al. [41], with some modifications. In this test, individual mice were forced to swim in a vertical transparent cylinder (30 cm in height and 12 cm in diameter) containing tap water at 23 ± 1 °C with 20 cm of depth. After 1 min of habituation, the immobility time of mice was recorded for a further 5 min. The mouse was considered immobile when it remained floating motionless in the water, making only the movements necessary to maintain the head above the water. A decrease in the duration of immobility is indicative of an antidepressant-like effect [41,42].

#### 2.5.4. Hippocampus Weight and Tissue Preparation

The hippocampus of mice was removed and weighed using an analytical balance. The result was expressed as the relative hippocampus weight (hippocampus weight/mouse body weight). Then, the hippocampus samples were homogenised in 50 mM Tris HCl at pH 7.4, 1:5 (*w*/*v*), and centrifuged (2500× *g*, 10 min) to yield a low-speed supernatant fraction (S1). Freshly prepared S1 was used for the determination of oxidative status and antioxidant defence parameters. 

#### 2.5.5. Reactive Species Quantification

The quantification of the RS levels in the hippocampus of mice was performed according to Loetchutinat et al. [43]. Briefly, an aliquot of S1 was incubated with 1 mM DCFH-DA and 50 mM Tris-HCl pH 7.4. The oxidation of DCHF-DA to fluorescent dichlorofluorescein was measured based on the fluorescence intensity emission at 520 nm (with 480 nm excitation). The results were expressed as arbitrary units of fluorescence (UF).

#### 2.5.6. Ferric Reducing Antioxidant Power (FRAP) Assay

The FRAP assay was performed according to Benzie and Strain [44]. This assay is based on the fact that reduction of a ferric tripyridyltriazine (Fe^III^-TPTZ) complex to the ferrous (Fe^II^-TPTZ) form at a low pH has an intense blue colour, which can be monitored by measuring the change in absorption at 593 nm. A standard curve of ascorbic acid was used, and the results were expressed as the μg equivalents of ascorbic acid.

#### 2.5.7. Quantification of Faecal LAB

The quantification of faecal LAB was performed as reported by Grimm et al. [45], with minor modifications. Briefly, the faecal pellets of all the mice (*n* = 6) were collected at the indicated time points (Days 0 and 28), pooled, mechanically homogenised in 0.85% NaCl solution (100 mg of faeces/mL) and diluted to 10^−8^. From each dilution, 100 µl of suspension was plated in triplicate on MRS agar (Merck^®^) and incubated for 24 h at 37 °C. After incubation, the agar plates were assessed for growth, the colonies were counted and the results were expressed as CFU/100 mg faeces.

### 2.6. Data Presentation and Statistical Analysis

All the results are given as the mean (s) ± standard error of means (S.E.M.). All in vitro tests were carried out in triplicate (except the antibiotic susceptibility test) and performed on the same day. The significance of the difference in mean values between more than two groups was evaluated by one-way analysis of variance (ANOVA) followed by post hoc multiple comparison by Tukey’s test. For in vivo experiments, data normality was evaluated by the D’Agostino and Pearson omnibus normality test. Parametric data were analysed using the Student’s *t*-test, and non-parametric data were analysed using the Mann–Whitney *U*-test. The level of *p* < 0.05 was considered as statistically significant. Statistical analysis was performed using the GraphPad Prism statistical software (version 6.01, GraphPad Software, USA).

## 3. Results and Discussion

### 3.1. Evaluation of Probiotic Characteristics

#### 3.1.1. Survival in Simulated Gastrointestinal Tract Conditions and Resistance to Different Concentrations of Salt (NaCl), pH and Bile Salts

An essential factor for the selection of a probiotic microorganism is the ability for it to survive and become active after entering the gastrointestinal tract. Thus, the strains must be able to survive at low pH and in the presence of digestive enzymes as well as adhere to epithelial cells to compete for the exclusion of pathogens [46]. In the evaluation of the gastric tract resistance in simulated form, it was possible to observe that the isolate was able to survive for up to 240 min in the presence of food (skimmed milk), as shown in Table 1. The presence of food and some food ingredients improved the viability of microorganisms during gastric transit; the proposed mechanism for this beneficial effect is linked to the increased pH of gastric content [47]. *Lactococcus lactis* LL95 maintained its viability above 8.2 Log CFU/mL. Even with a reduction of viable cells during in vitro simulation, the isolate may still be able to reach the intestine and promote beneficial effects if it reaches a density of around 6 Log CFU/mL [48].

One of the most important selection criteria for probiotics is the resistance to the low pH of gastric juice in the stomach and bile salts in the small intestine. Therefore, as probiotics are usually administrated orally, they must be able to survive passing through the stomach and small intestine. [8]. *Lactococcus lactis* LL95 was able to survive the different conditions of NaCl (4.5% and 6%) and bile salts (0.3% and 1%) and did not exhibit growth at pH 2. However, according to Huang and Adams [28], to survive in the gut, organisms must be tolerant to the pH of the stomach, which ranges from 2.5 to 3.5, and which can be as high as 4.5 after meals. Thus, tolerance to bile salts demonstrates that this strain is likely to be resistant to the conditions of the stomach and intestines.

#### 3.1.2. Autoaggregation and Coaggregation Abilities

Aggregation ability has been suggested to be an important property of many bacterial strains used as probiotics [49]. The autoaggregation ability of *L. lactis* LL95 and *L. monocytogenes* ATCC 7644 is presented in Table 2. Collado et al. [30] showed that the autoaggregation of lactic acid bacteria is correlated to their adhesion ability. The coaggregation levels of probiotic bacteria with *L. monocytogenes* ATCC 7644 are shown in Table 3. The best results were observed at 37 °C and after 24 h incubation. Coaggregation of probiotic bacteria with potential gut pathogens could contribute to the positive properties of this strain. This plays a significant role, especially in the human gut, in the prevention of colonisation by pathogenic microorganisms [50]. It has been previously reported that coaggregation abilities may allow LAB strains to inhibit the growth of pathogenic strains in the gastrointestinal tract [51]. Moreover, LAB strains were found to control a microenvironment around the pathogens and increase the concentration of excreted antimicrobial substances in the process of coaggregation [50].

#### 3.1.3. Antimicrobial Activity

*Lactococcus lactis* LL95 showed antimicrobial activity only against *L. monocytogenes* ATCC 7644, with an inhibition halo of 12 mm. The antimicrobial activity of probiotic bacteria is attributed to the production of metabolites, such as lactic and acetic acid, hydrogen peroxide, ethanol and other compounds with antimicrobial activity, beyond the bacteriocins [52]. In this respect, the most important and best characterised antimicrobials produced by LAB are lactic and acetic acid and the toxic effects of these organic acids include the reduction of intracellular pH [53]. As such, we believe that the antimicrobial activity of *L. lactis* LL95 could result from an increased rate of acidification.

#### 3.1.4. Antibiotic Susceptibility Tests

The antibiotic resistance of microorganisms is an extremely important element to demonstrate the safety of new probiotic cultures [5]. *Lactococcus lactis* LL95 showed phenotypic resistance to amikacin, trimethoprim-sulphamethoxazole, tetracycline, clindamycin and sulphonamide (Table 4). 

Antibiotic-resistant *Lactococcus strains* may be selected for environments where they are exposed to antibiotics, such as the udder of antibiotic-treated cows and, therefore, the products derived from their raw milk [54]. Once that some food and certain food supplements can introduce large numbers of live *L. lactis* into the human and animal gastrointestinal tract [55], there is the concern about the possibility of commensal bacteria acquiring antibiotic resistance genes from their environment, since this resistance could be transferred to pathogenic species, which would hamper the treatment of infections [56]. To prevent potential antibiotic resistance traits from being transferred to human or animal commensal microbiota and to pathogenic bacteria, the antimicrobial susceptibility of food-associated LAB, such as *L. lactis* strains, should be determined [57]. *Lactococcus lactis* LL95 showed sensibility to ampicillin, chloramphenicol, erythromycin, penicillin, vancomycin and ciprofloxacin (Table 4). Gad et al. demonstrated that *Lactococcus* spp. was susceptible to vancomycin and chloramphenicol and showed high resistance to tetracycline [58]. According to Teuber et al. [59], antibiotic susceptibility to ampicillin, chloramphenicol, erythromycin, gentamicin, penicillin and vancomycin is a common intrinsic characteristic of *L. lactis*.

### 3.2. In Vitro Antioxidant Activity Experiments

#### Determination of In Vitro Antioxidant Activities of *Lactococcus lactis* subsp. *cremoris* LL95

The in vitro assays for the determination of antioxidant activity were based on the determination of the scavenging capacity against reactive oxidative species (ROS), such as superoxide anion scavenging assay, hydrogen peroxide scavenging assay and scavenging activity against stable non-biological radicals. In this study, lyophilised cells exhibited higher antioxidant activity in the in vitro assays compared to intact and heat-killed cells (Table 5). Thus, we selected lyophilised cells for the in vivo study. 

Probiotics are known to have many beneficial health effects, and their consumption shows that strain-specific probiotics can present antioxidant activity and reduce damage caused by oxidation [60]. The underlying mechanism of the oxidation-resistant ability of probiotics is not yet fully understood. One of the proposed mechanisms is metal ion chelating ability, as demonstrated by Lin and Yen [61] and Lee et al. [62]. Moreover, the metabolic antioxidant activity of LAB may result from their own antioxidant enzymes (superoxide dismutase and catalase), the production of antioxidant metabolites (folate and glutathione), the up-regulation of the antioxidant activities of the host, increasing the levels of antioxidant metabolites of the host, the regulation of signalling pathways, the down-regulation activities of enzymes producing ROS and the regulation of intestinal microbiota [60,63].

### 3.3. In Vivo Experiments

All mice receiving *L. lactis* LL95 remained healthy throughout the study. There were no signs of diarrhoea, weight loss or loss of appetite. Animal gain weight was not affected by probiotic treatment. The number of *L. lactis* LL95 CFU per 100 µL were determined throughout the study to ensure that the appropriate concentration was present in the aliquots of the probiotic preparation. The viability did not vary during the study period (data not shown).

#### 3.3.1. *Lactococcus lactis* subsp. *cremoris* LL95 Had No Effect on Locomotor Activity and Reduced the Anxiety-Like Behaviour

**Figure 2:**
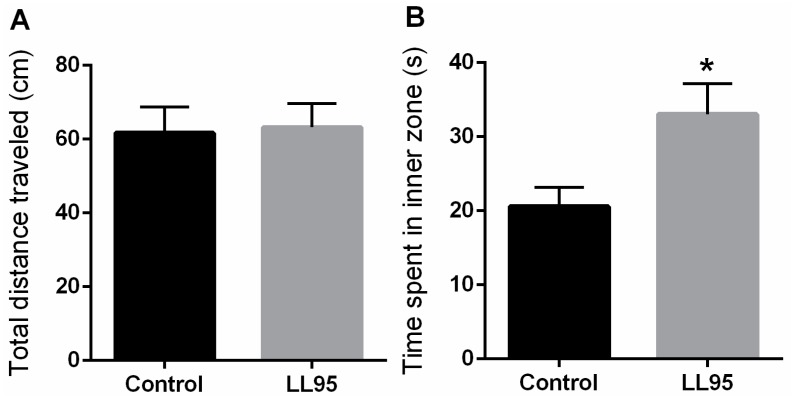

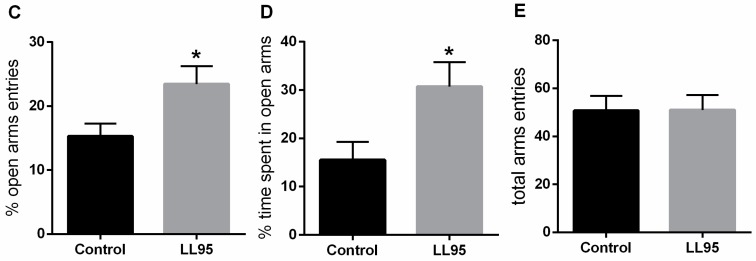
Effect of *L. lactis* LL95 on locomotor activity and anxiety-like behaviour in the OFT and EPM. There was no difference in the distance travelled (**A**) between groups in the OFT. *L. lactis* LL95 induced an increase on the time spent in inner zone than control group (**B**). *L. lactis* LL95 induced an increased in per cent of open arms entries and per cent time spent in the open arms (**C**,**D**). The total number of entries did not differ between groups (**E**). Data are expressed as mean ± S.E.M. (*n* = 6). * *p* < 0.05 vs. control group.

The administration of LL95 did not affect locomotor activity in the OFT (*t* = 0.6722, df = 10; *p* = 0.5167; Figure 2A) and in the EPM (*t* = 0.038, df = 10; *p* = 0.9704; Figure 2E). As shown in Figure 2B, the LL95 group remained more time in the inner zone (*t* = 2.556, df = 10; *p* = 0.0285) than the control group. In the EPM, groups differed in the percentage of entries and time spent (Figure 2C,D) in the open arms (*t* = 2.391, df = 10, *p* = 0.0379 and *t* = 2.396, df = 10; *p* = 0.0376, respectively), suggesting anxiolytic effects. 

Mice that spent significantly more time exploring the unprotected central area in the OFT apparatus demonstrated anxiolytic-like baseline behaviour, since rodents typically spend a significantly greater amount of time exploring the periphery of the arena, usually in contact with the walls [64]. Moreover, an increased activity in the open arms in the EPM also reflects an anxiolytic-like behaviour [65]. The premise that basic physiological mechanisms underlying fear in rodents can be equated to similar mechanisms operating in humans provides a degree of validity to these paradigms [66]. 

Several studies have demonstrated the beneficial effects of modulation of the microbiota by the use of probiotics on behaviour and brain chemistry [67]. For instance, Bravo et al. [10] showed that chronic treatment with *Lactobacillus rhamnosus* (JB-1) reduced stress-induced corticosterone and anxiety-like behaviour in normal, healthy animals. In animals subjected to 21 days of restraint stress, *Lactobacillus helveticus* NS8 supplementation increased time spent in the open arms in EPM [14]. Similarly, a recent study shows that animals that received a probiotics treatment exhibited anxiolytic-like behaviours after exposure to an unpredictable chronic mild stress (UCMS) test for four weeks [68]. Moreover, several clinical studies have demonstrated significant health benefits after consumption of probiotics, including decreased anxiety ratings and a lowered stress response in healthy volunteers, reinforcing the benefits of probiotic supplementation in the treatment of mood- and stress-related diseases [69,70,71].

#### 3.3.2. *Lactococcus lactis* subsp. *cremoris* LL95 Reduces Depressive-Like Behaviour in Mice 

**Figure 3:**
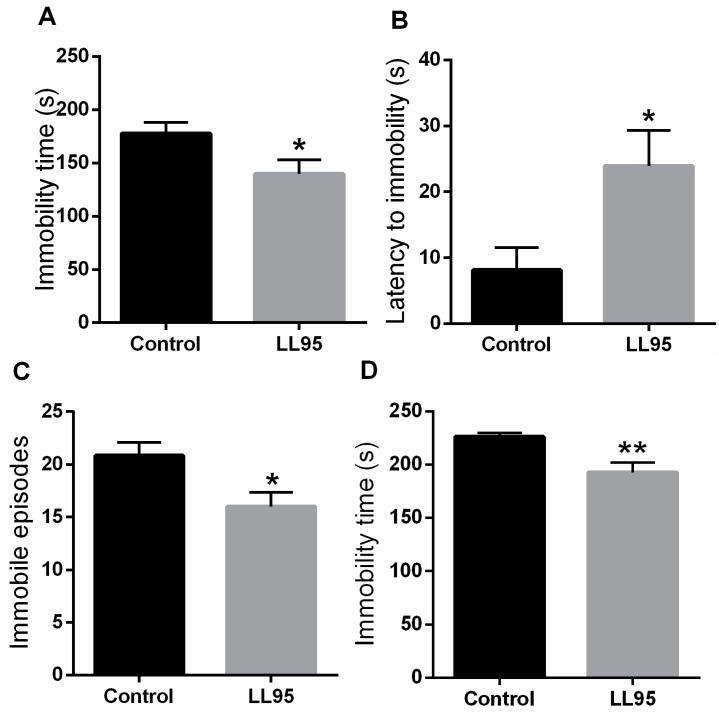
Effect of *L. lactis* LL95 on depressive-like behaviour in the TST and FST. *L. lactis* LL95 induced a decrease in the immobility time (**A**), an increase to the latency for first episode of immobility (**B**), and a decrease on immobile episodes (**C**) in TST. *L. lactis* LL95 induced a decrease in the immobility time in FST (**D**). Data are expressed as mean ± S.E.M. (*n* = 6). * *p* < 0.05, ** *p* < 0.01 vs. control group.

The immobility time in the TST and FST in animals fed with *L. lactis* LL95 is shown in Figure 3A,D, respectively. Treatment with *L. lactis* LL95 resulted in a decrease in immobility time when compared to the control group in the TST (*t* = 2.232, df = 10; *p* = 0.0497) and FST (*t* = 3.316, df = 10; *p* = 0.0078). Moreover, chronic administration of LL95 was able to increase the latency to immobility (*t* = 2.474, df = 10; *p* = 0.0329; Figure 3B) and decrease the number of immobile episodes (*t* = 2.611, df = 10, *p* = 0.0260; Figure 3C) in the TST. 

It is believed that intestinal bacteria play a major role in the bidirectional signalling between the brain and the gut. Therefore, several studies have explored the link between gut microbiota and mood disorders, investigating the role of the gut–brain axis in the pathophysiology of depression [72]. Supporting our study, Bravo et al. [10] showed that *L. rhamnosus* (JB-1)-fed animals spent significantly less time immobile in the FST, suggesting that non-pathogenic bacteria may have beneficial effects in the treatment of depression. Furthermore, it was shown that chronic consumption of probiotic *Bifidobacterium infantis* normalised the effects of maternal separation on immobility in the FST [13]. In addition, it has been shown that, independently of diet, probiotic treatment could reduce depressive-like behaviour in the FST by 34% [17]. Akkasheh et al. [73] showed that probiotic administration in patients with major depression disorder for eight weeks had a beneficial effect on the Beck Depression Inventory and in certain metabolic profiles and biomarkers of oxidative stress.

#### 3.3.3. *Lactococcus lactis* subsp. *cremoris* LL95 Increased Hippocampal Weight and Prevented Oxidative Stress in the Hippocampus in Mice 

**Figure 4:**
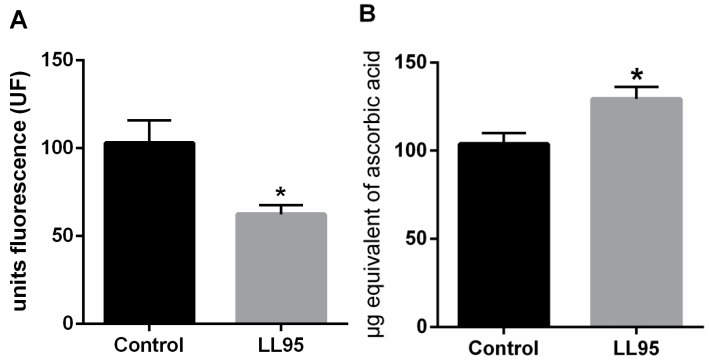
Effect of *L. lactis* LL95 in oxidative stress in hippocampus. *L. lactis* LL95 induced a decrease on levels of reactive species (**A**) and an increase in the ferric reducing/antioxidant power (**B**) on hippocampus in mice. Data are expressed as mean ± S.E.M. (n=6). * *p* < 0.05 vs. control group.

The weight of the hippocampus increased by 33% in female mice fed with *L. lactis* LL95 (control vs. LL95: 0.967 ± 0.109 vs. 1.467 ± 0.171; *p* = 0.0330). We observed a significant decrease in hippocampal RS levels in LL95-fed mice when compared with the control group (control vs. LL95: 103.1 ± 12.95 vs. 62.38 ± 5.27; *p* = 0.0152; Figure 4A). In addition, the chronic administration of *L. lactis* LL95 caused an increase in the FRAP assay (*t* = 2.773, df = 10; *p* = 0.0197; Figure 4B). 

Several mechanisms are suggested as responsible for the biological activities of probiotics, one of the most debated being antioxidant activity [60]. In fact, among the beneficial effects of probiotics, protection against oxidative stress in humans and in animal models has been previously reported [74,75,76]. From this perspective, the selection of specific strains and the evidence of their effectiveness, resulting in the control of reactive species, can be exploited to formulate novel probiotic foods or supplements that can exert a role in the prevention of oxidative stress and related diseases.

#### 3.3.4. Administration of *Lactococcus lactis* subsp. *cremoris* LL95 Increased the Faecal Content of LAB

**Figure 5:**
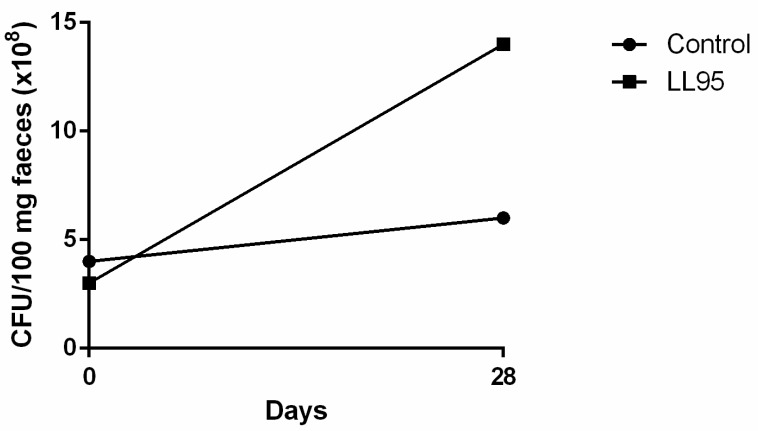
Effect of *L. lactis* LL95 on faecal content of LAB. The administration of *L. lactis* LL95 induced an increase on faecal content of LAB after 28 days in mice. Data are expressed as CFU/100 mg faeces.

As an indirect way to identify the presence of LAB in faeces, the quantification was performed in faecal pellets through plating on MRS agar. Faecal LAB counts were similar in both groups before the start of *L. lactis* LL95 administration (Figure 5). On the 28th day, we observed that the *L. lactis* LL95 group showed an increased number of LAB CFU per 100 mg of faeces when compared to the control group (control vs. LL95: 6.0 × 10^8^ vs. 1.4 × 10^9^). 

The faecal LAB count in the *L. lactis* LL95-fed group was about three times greater than that of the control group, indicating the survival of the bacteria in the gastrointestinal tract. Strains that are able to act as probiotics and can exert a protective action against infections have gained increased interest. LAB are beneficial bacteria normally associated with a balanced normal gut microbiota. They have an important role in the maintenance of health by producing antimicrobial substances, such as lactic acid, that act against pathogens, or competing for cell-surface [77]. Hence, the modulation of the microbiota gut can affect many aspects of physiology, including behaviour disorders, as demonstrated by many studies [10,13,78]. Thus, we believe that the high levels of LAB microbiota in the gut in the probiotic-fed group may be responsible for the positive results presented here. Evidence of the gut–brain axis and the effect of modulation of gut microbiota on mental health has shown new perspectives in determining the causative agents and possible treatments of depression- and anxiety-like disorders. Nonetheless, continuous research in this area is continuing to prove that these effects are mostly strain-specific and should not be generalised.

## 4. Conclusions

Our findings suggest that *L. lactis* LL95 may be a potential probiotic and showed efficacy in exerting a free-radical scavenging effect. More importantly, the data of our mouse model clearly suggest the propensity of oral supplementation of *L. lactis* LL95 to improve depressive- and anxiety-like behaviour and the antioxidant parameters in the hippocampus. Although the underlying molecular mechanisms require further study, it is reasonable to speculate that the intake of this potential probiotic strain is likely to confer protective benefits in vivo against oxidative damage-mediated neurodegenerative conditions. However, we cannot exclude the possible involvement of other functional processes and systems that may contribute to the beneficial effects of *L. lactis* LL95. 

## Figures and Tables

**Table 1:** Viable cells of *Lactococcus lactis* subsp. *cremoris* LL95 when submitted to simulated gastric transit.

Time (min)	Log CFU/mL	
	SS	SM
0	8.22 ± 0.01 ^a^	8.27 ± 0.02 ^b^
15	8.45 ± 0.04 ^a^	8.30 ± 0.00 ^b^
30	8.23 ± 0.00 ^a^	8.38 ± 0.13 ^a^
60	7.57 ± 0.11 ^a^	8.27 ± 0.18 ^b^
120	5.43 ± 0.18 ^a^	8.46 ± 0.11 ^b^
180	2.93 ± 0.31 ^a^	8.18 ± 0.00 ^b^
240	0.57 ± 0.76 ^a^	8.25 ± 0.10 ^b^

The presented values are the means of three determinations ± S.E.M. Means within the same row with different letters differ significantly (*p* < 0.05). SS: saline; SM: skim milk.

**Table 2:** Autoaggregation (%) of *Lactococcus lactis* subsp. *cremoris* LL95 and *Listeria monocytogenes* ATCC 7644 during different incubation periods and temperatures.

Time (h)	LL95		LM	
	20 °C	37 °C	20 °C	37 °C
2	22.13 ± 0.12 ^a^	24.10 ± 0.14 ^b^	12.00 ± 0.15 ^a^	19.26 ± 0.31 ^b^
20	11.70 ± 0.14 ^a^	16.80 ± 0.52 ^b^	20.18 ± 0.31 ^a^	22.72 ± 0.31 ^b^
24	15.18 ± 0.13 ^a^	21.32 ± 0.28 ^a^	18.22 ± 0.15 ^a^	27.10 ± 0.31 ^b^

LL95: *Lactococcus lactis* subsp. *cremoris* LL95; LM, *Listeria monocytogenes* ATCC 7644. The presented values are the means of three determinations ± S.E.M. Means within the same row with different letters differ significantly (*p* < 0.05).

**Table 3:** Coaggregation (%) ability of *Lactococcus lactis* subsp. *cremoris* LL95 to *Listeria monocytogenes* ATCC 7644 during different incubation periods and temperatures.

Time (h)	20 °C	37 °C
2	7.90 ± 0.42 ^a^	0 ± 1.98 ^b^
20	4.95 ± 0.08 ^a^	12.73 ± 2.62 ^b^
24	0 ± 5.49 ^a^	18.86 ± 4.44 ^b^

The presented values are the means of three determinations ± S.E.M. Means within the same row with different letters differ significantly (*p* < 0.05).

**Table 4:** Antibiotic susceptibility of *Lactococcus lactis* subsp. *cremoris* LL95.

Antibiotics	Inhibition Zone Diameters (mm)	Classification ^1^
Ampicillin	23	Sensitive
Chloramphenicol	31	Sensitive
Erythromycin	23	Sensitive
Penicillin	23	Sensitive
Vancomycin	22	Sensitive
Ciprofloxacin	29	Sensitive
Gentamicin	16	Intermediate sensitivity
Amikacin	15	Resistant
Trimethoprim-sulphamethoxazole	0	Resistant
Tetracycline	15	Resistant
Clindamycin	9	Resistant
Sulphonamide	0	Resistant

^1^ The interpretive criteria for the diameters of inhibition zones were those described by European Food Safety Authority [25].

**Table 5:** In vitro antioxidant activities of *Lactococcus lactis* subsp. *cremoris* LL 95 on DPPH, ABTS^•+^ and FRAP assays.

LL 95 cells	DPPH (U/mL)	ABTS^•+^ (%)	FRAP (mM)
Intact cells	1.30 ± 0.50 ^a^	56.50 ± 4.68 ^a^	22.40 ± 0.39 ^a^
Heat-killed cells	6.85 ± 0.45 ^a,b^	32.34 ± 0.22 ^b^	13.49 ± 0.49 ^b^
Lyophilised cells	9.40 ± 1.90 ^b^	79.07 ± 4.73 ^a^	45.04 ± 0.04 ^c^

The presented values are the means of three determinations ± S.E.M. Means within the same column with different letters differ significantly (*p* < 0.05).

## References

[1] Bischoff S.C., Barbara G., Buurman W., Ockhuizen T., Schulzke J.-D., Serino M., Tilg H., Watson A., Wells J.M. (2014). Intestinal permeability—A new target for disease prevention and therapy. BMC Gastroenterol..

[2] Carding S., Verbeke K., Vipond D.T., Corfe B.M., Owen L.J. (2015). Dysbiosis of the gut microbiota in disease. Microb. Ecol. Health Dis..

[3] DeGruttola A.K., Low D., Mizoguchi A., Mizoguchi E. (2016). Current understanding of dysbiosis in disease in human and animal models. Inflamm. Bowel Dis..

[4] Natividad J.M., Verdu E.F. (2013). Modulation of intestinal barrier by intestinal microbiota: Pathological and therapeutic implications. Pharmacol. Res..

[5] FAO, WHO (2001). Joint FAO/WHO Expert Consultation on Evaluation of Health and Nutritional Properties of Probiotics in Food Including Powder Milk with Live Lactic Acid Bacteria.

[6] Reis J.A., Paula A.T., Casarotti S.N., Penna A.L.B. (2012). Lactic Acid Bacteria Antimicrobial Compounds: Characteristics and Applications. Eng. Rev..

[7] Saad N., Delattre C., Urdaci M., Schmitter J., Bressollier P. (2013). An overview of the last advances in probiotic and prebiotic field. LWT Food Sci. Technol..

[8] Pereira G.V.D.M., Coelho B.D.O., Júnior A.I.M., Thomaz-Soccol V., Soccol C.R. (2018). How to select a probiotic? A review and update of methods and criteria. Biotechnol. Adv..

[9] Dinan T.G., Cryan J.F. (2017). Brain-Gut-Microbiota Axis and Mental Health. Psychosom. Med..

[10] Bravo J.A., Forsythe P., Chew M.V., Escaravage E., Savignac H.M., Dinan T.G., Bienenstock J., Cryan J.F. (2011). Ingestion of Lactobacillus strain regulates emotional behavior and central GABA receptor expression in a mouse via the vagus nerve. Proc. Natl. Acad. Sci. USA.

[11] Cryan J.F., Dinan T.G. (2012). Mind-altering microorganisms: The impact of the gut microbiota on brain and behaviour. Nat. Rev. Neurosci..

[12] Desbonnet L., Clarke G., Shanahan F., Dinan T.G., Cryan J.F., Dinan T., Cryan J. (2013). Microbiota is essential for social development in the mouse. Mol. Psychiatry.

[13] Desbonnet L., Garrett L., Clarke G., Kiely B., Cryan J., Dinan T., Dinan T. (2010). Effects of the probiotic Bifidobacterium infantis in the maternal separation model of depression. Neuroscience.

[14] Liang S., Wang T., Hu X., Luo J., Li W., Wu X., Duan Y., Jin F. (2015). Administration of Lactobacillus helveticus NS8 improves behavioral, cognitive, and biochemical aberrations caused by chronic restraint stress. Neuroscience.

[15] Liu Y.-W., Liu W.-H., Wu C.-C., Juan Y.-C., Wu Y.-C., Tsai H.-P., Wang S., Tsai Y.-C. (2016). Psychotropic effects of Lactobacillus plantarum PS128 in early life-stressed and naïve adult mice. Brain Res..

[16] Savignac H.M., Kiely B., Dinan T., Cryan J.F. (2014). Bifidobacteria exert strain-specific effects on stress-related behavior and physiology in BALB/c mice. Neurogastroenterol. Motil..

[17] Abildgaard A., Elfving B., Hokland M., Wegener G., Lund S. (2017). Probiotic treatment reduces depressive-like behaviour in rats independently of diet. Psychoneuroendocrinology.

[18] Abdrabou A.M., Osman E.Y., Aboubakr O.A. (2018). Comparative therapeutic efficacy study of Lactobacilli probiotics and citalopram in treatment of acute stress-induced depression in lab murine models. Hum. Microbiome J..

[19] WHO (2017). Depression and Other Common Mental Disorders: Global Health Estimates.

[20] Zhou Y., Cao Z., Yang M., Xi X., Guo Y., Fang M., Cheng L., Du Y. (2017). Comorbid generalized anxiety disorder and its association with quality of life in patients with major depressive disorder. Sci. Rep..

[21] Anderson H.D., Pace W.D., Libby A.M., West D.R., Valuck R.J. (2012). Rates of 5 Common Antidepressant Side Effects Among New Adult and Adolescent Cases of Depression: A Retrospective US Claims Study. Clin. Ther..

[22] Vlieg J.E.V.H., Rademaker J.L., Bachmann H., Molenaar D., Kelly W.J., Siezen R.J. (2006). Natural diversity and adaptive responses of *Lactococcus lactis*. Curr. Opin. Biotechnol..

[23] Kosaka A., Yan H., Ohashi S., Gotoh Y., Sato A., Tsutsui H., Kaisho T., Toda T., Tsuji N.M. (2012). *Lactococcus lactis* subsp. *cremoris* FC triggers IFN-γ production from NK and T cells via IL-12 and IL-18. Int. Immunopharmacol..

[24] Maruo T., Gotoh Y., Nishimura H., Ohashi S., Toda T., Takahashi K. (2012). Oral administration of milk fermented with *Lactococcus lactis* subsp. *cremoris* FC protects mice against influenza virus infection. Lett. Appl. Microbiol..

[25] Hamed H., Chaari F., Ghannoudi Z., Dhouib K., Chaabouni S., El Feki A., Gargouri A. (2018). Fermented camel milk by *Lactococcus lactis* subsp. *cremoris* attenuates erythrocytes oxidative stress-induced hematological and immunological damage in CCl4-intoxicated mice. Environ. Sci. Pollut. Res..

[26] Hamed H., Chaari F., Ghannoudi Z., Elfeki A., Ellouz S.C., Gargouri A. (2018). Beneficial effects of fermented camel milk by *Lactococcus lactis* subsp. *cremoris* on cardiotoxicity induced by carbon tetrachloride in mice. Biomed. Pharmacother..

[27] Figueroa-González I., Quijano G., Ramirez G., Cruz-Guerrero A., Figueroa-González I., Cruz-Guerrero A. (2011). Probiotics and prebiotics-perspectives and challenges. J. Sci. Agric..

[28] Huang Y., Adams M.C. (2004). In vitro assessment of the upper gastrointestinal tolerance of potential probiotic dairy propionibacteria. Int. J. Microbiol..

[29] Drosinos E., Mataragas M., Xiraphi N., Moschonas G., Gaitis F., Metaxopoulos J. (2005). Characterization of the microbial flora from a traditional Greek fermented sausage. Meat Sci..

[30] Collado M.C., Meriluoto J., Salminen S. (2008). Adhesion and aggregation properties of probiotic and pathogen strains. Eur. Food Res. Technol..

[31] Biscola V., Todorov S., Capuano V., Abriouel H., Gálvez A., Franco B. (2013). Isolation and characterization of a nisin-like bacteriocin produced by a *Lactococcus lactis* strain isolated from charqui, a Brazilian fermented, salted and dried meat product. Meat Sci..

[32] European Food Safety Authority (2003). Opnion of the Scientific Committee on Animal Nutrition on the criteria for assessing the safety of micro-organisms resistant to antibiotics of human clinical and veterinary importance. Eur. Food Saf. Auth. J..

[33] Liasi S.A., Azmi T.I., Hassan M.D., Shuhaimi M., Rosfarizan M., Ariff A.B. (2009). Antimicrobial activity and antibiotic sensitivity of three isolates of lactic acid bacteria from fermented fish product, Budu. Malays. J. Microbiol..

[34] Lim L.H., Li H.Y., Huang C.H., Chua K.Y., Lee B.W., Lee Y.K. (2008). The Effects of Heat-Killed Wild-Type Lactobacillus casei Shirota on Allergic Immune Responses in an Allergy Mouse Model. Int. Arch. Immunol..

[35] Xing J., Wang G., Zhang Q., Liu X., Gu Z., Zhang H., Chen Y.Q., Chen W. (2015). Determining Antioxidant Activities of Lactobacilli Cell-Free Supernatants by Cellular Antioxidant Assay: A Comparison with Traditional Methods. PLoS ONE.

[36] Han Q., Kong B., Chen Q., Sun F., Zhang H. (2017). In vitro comparison of probiotic properties of lactic acid bacteria isolated from Harbin dry sausages and selected probiotics. J. Funct. Foods.

[37] Parker G., Brotchie H. (2010). Gender differences in depression. Int. Rev..

[38] Seibenhener M.L., Wooten M.C. (2015). Use of the Open Field Maze to Measure Locomotor and Anxiety-like Behavior in Mice. J. Vis. Exp..

[39] Lister R.G. (1987). The use of a plus-maze to measure anxiety in the mouse. Psychopharmacology.

[40] Steru L., Chermat R., Thierry B., Simon P. (1985). The tail suspension test: A new method for screening antidepressants in mice. Psychopharmacology.

[41] Porsolt R.D., Le Pichon M., Jalfre M. (1977). Depression: A new animal model sensitive to antidepressant treatments. Nature.

[42] Cryan J.F., Markou A., Lucki I. (2002). Assessing antidepressant activity in rodents: Recent developments and future needs. Pharmacol. Sci..

[43] Loetchutinat C., Kothan S., Dechsupa S., Meesungnoen J., Jay-Gerin J.-P., Mankhetkorn S. (2005). Spectrofluorometric determination of intracellular levels of reactive oxygen species in drug-sensitive and drug-resistant cancer cells using the 2′,7′-dichlorofluorescein diacetate assay. Radiat. Phys. Chem..

[44] Benzie I.F., Strain J. (1999). Ferric reducing/antioxidant power assay: Direct measure of total antioxidant activity of biological fluids and modified version for simultaneous measurement of total antioxidant power and ascorbic acid concentration. Methods Enzymol..

[45] Grimm V., Radulović K., Riedel C.U. (2015). Colonization of C57BL/6 Mice by a Potential Probiotic Bifidobacterium bifidum Strain under Germ-Free and Specific Pathogen-Free Conditions and during Experimental Colitis. PLoS ONE.

[46] Maragkoudakis P.A., Zoumpopoulou G., Miaris C., Kalantzopoulos G., Pot B., Tsakalidou E. (2006). Probiotic potential of Lactobacillus strains isolated from dairy products. Int. J..

[47] Monteagudo-Mera A., Rodríguez-Aparicio L.B., Rúa J., Martínez-Blanco H., Navasa N., García-Armesto M.R., Ferrero M. (2012). Ángel In vitro evaluation of physiological probiotic properties of different lactic acid bacteria strains of dairy and human origin. J. Funct. Foods.

[48] Charteris W.P., Kelly P.M., Morelli L., Collins J.K. (1998). Ingredient selection criteria for probiotic microorganisms in functional dairy foods. Int. J. Technol..

[49] Sgorbati B., Palenzona D., Del Re B., Miglioli M. (2000). Adhesion, autoaggregation and hydrophobicity of 13 strains of Bifidobacterium longum. Lett. Appl. Microbiol..

[50] Kaewnopparat S., Dangmanee N., Kaewnopparat N., Srichana T., Chulasiri M., Settharaksa S. (2013). In vitro probiotic properties of Lactobacillus fermentum SK5 isolated from vagina of a healthy woman. Anaerobe.

[51] Botes M., Loos B., Van Reenen C.A., Dicks L.M.T., Reenen C.A. (2008). Adhesion of the probiotic strains Enterococcus mundtii ST4SA and Lactobacillus plantarum 423 to Caco-2 cells under conditions simulating the intestinal tract, and in the presence of antibiotics and anti-inflammatory medicaments. Arch. Microbiol..

[52] Vandenbergh P.A. (1993). Lactic acid bacteria, their metabolic products and interference with microbial growth. FEMS Microbiol. Rev..

[53] Leroy F., Verluyten J., De Vuyst L. (2006). Functional meat starter cultures for improved sausage fermentation. Int. J. Microbiol..

[54] Bennish M.L. (1999). Animals, humans, and antibiotics: Implications of the veterinary use of antibiotics on human health. Adv. Pediatr. Infect. Dis..

[55] Guarner F., Malagelada J.-R. (2003). Gut flora in health and disease. Lancet.

[56] Hamilton-Miller J. (2004). Antibiotic resistance from two perspectives: Man and microbe. Int. J. Antimicrob. Agents.

[57] Armas F., Camperio C., Marianelli C. (2017). In Vitro Assessment of the Probiotic Potential of *Lactococcus lactis* LMG 7930 against Ruminant Mastitis-Causing Pathogens. PLoS ONE.

[58] Gad G.F.M., Abdel-Hamid A.M., Farag Z.S.H. (2014). Antibiotic resistance in lactic acid bacteria isolated from some pharmaceutical and dairy products. Braz. J. Microbiol..

[59] Teuber M., Meile L., Schwarz F., Veld J.H.J.H. (1999). Acquired antibiotic resistance in lactic acid bacteria from food. Lactic Acid Bact. Genet. Metab. Appl..

[60] Wang Y., Wu Y., Wang Y., Xu H., Mei X., Yu D., Wang Y., Li W. (2017). Antioxidant Properties of Probiotic Bacteria. Nutrients.

[61] Lin M.-Y., Yen C.-L. (1999). Antioxidative Ability of Lactic Acid Bacteria. J. Agric. Chem..

[62] Lee J., Hwang K.-T., Chung M.-Y., Cho D.-H., Park C.-S. (2005). Resistance of Lactobacillus casei KCTC 3260 to Reactive Oxygen Species (ROS): Role for a Metal Ion Chelating Effect. J. Sci..

[63] Li Y., Hugenholtz J., Abee T., Molenaar D. (2003). Glutathione Protects *Lactococcus lactis* against Oxidative Stress. Appl. Environ. Microbiol..

[64] Bailey K.R., Crawley J.N. (2009). Anxiety-Related Behaviors in Mice. Methods of Behavior Analysis in Neuroscience.

[65] Walf A., Frye C. (2007). The use of the elevated plus maze as an assay of anxiety-related behavior in rodents. Nat. Protoc..

[66] Cryan J.F., Holmes A. (2005). The ascent of mouse: Advances in modelling human depression and anxiety. Nat. Rev. Drug Discov..

[67] Slyepchenko A., Carvalho A.F., Cha D.S., Kasper S., McIntyre R.S. (2014). Gut emotions - mechanisms of action of probiotics as novel therapeutic targets for depression and anxiety disorders. CNS Neurol. Disord. Drug Targets.

[68] Avolio E., Fazzari G., Zizza M., De Lorenzo A., Di Renzo L., Alo R., Facciolo R.M., Canonaco M. (2018). Probiotics modify body weight together with anxiety states via pro-inflammatory factors in HFD-treated Syrian golden hamster. Behav. Brain Res..

[69] Messaoudi M., LaLonde R., Violle N., Javelot H., Desor D., Nejdi A., Bisson J.-F., Rougeot C., Pichelin M., Cazaubiel M. (2010). Assessment of psychotropic-like properties of a probiotic formulation (*Lactobacillus helveticus* R0052 and *Bifidobacterium longum* R0175) in rats and human subjects. Br. J. Nutr..

[70] Steenbergen L., Sellaro R., Van Hemert S., Bosch J.A., Colzato L.S. (2015). A randomized controlled trial to test the effect of multispecies probiotics on cognitive reactivity to sad mood. Brain Behav. Immun..

[71] Mohammadi A.A., Jazayeri S., Khosravi-Darani K., Solati Z., Mohammadpour N., Asemi Z., Adab Z., Djalali M., Tehrani-Doost M., Hosseini M. (2015). The effects of probiotics on mental health and hypothalamic–pituitary–adrenal axis: A randomized, double-blind, placebo-controlled trial in petrochemical workers. Nutr. Neurosci..

[72] Schmidt C. (2015). Thinking from the Gut. Nature.

[73] Akkasheh G., Kashani-Poor Z., Tajabadi-Ebrahimi M., Jafari P., Akbari H., Taghizadeh M., Memarzadeh M.R., Asemi Z., Esmaillzadeh A., Information P.E.K.F.C. (2016). Clinical and metabolic response to probiotic administration in patients with major depressive disorder: A randomized, double-blind, placebo-controlled trial. Nutrition.

[74] Amaretti A., Nunzio M., Pompei A., Raimondi S., Rossi M., Bordoni A., Di Nunzio M. (2012). Antioxidant properties of potentially probiotic bacteria: In vitro and in vivo activities. Appl. Microbiol. Biotechnol..

[75] Sun J., Hu X.-L., Le G.-W., Shi Y.-H. (2012). Inhibition of Fe-induced colon oxidative stress by lactobacilli in mice. J. Microbiol. Biotechnol..

[76] Songisepp E., Kals J., Kullisaar T., Mändar R., Hütt P., Zilmer M., Mikelsaar M. (2005). Evaluation of the functional efficacy of an antioxidative probiotic in healthy volunteers. Nutr. J..

[77] Liu W., Ren P., He S., Xu L., Yang Y., Gu Z., Zhou Z. (2013). Comparison of adhesive gut bacteria composition, immunity, and disease resistance in juvenile hybrid tilapia fed two different Lactobacillus strains. Fish Shellfish. Immunol..

[78] Kelly J.R., Borre Y., Brien C.O., Patterson E., El Aidy S., Deane J., Kennedy P.J., Beers S., Scott K., Moloney G. (2016). Transferring the blues: Depression-associated gut microbiota induces neurobehavioural changes in the rat. J. Psychiatr. Res..

